# Pretransplant endotrophin predicts delayed graft function after kidney transplantation

**DOI:** 10.1038/s41598-022-07645-y

**Published:** 2022-03-08

**Authors:** Martin Tepel, Firas F. Alkaff, Daan Kremer, Stephan J. L. Bakker, Olivier Thaunat, Subagini Nagarajah, Qais Saleh, Stefan P. Berger, Jacob van den Born, Nicoline V. Krogstrup, Marie B. Nielsen, Rikke Nørregaard, Bente Jespersen, Nadja Sparding, Federica Genovese, Morten A. Karsdal, Daniel G. K. Rasmussen

**Affiliations:** 1 Department of Nephrology, Odense University Hospital, Odense, Denmark; 2 Institute of Molecular Medicine, Cardiovascular and Renal Research, University of Southern Denmark, J.B. Winsløwsvej 21.3, 5000 Odense C, Denmark; 3 Division of Nephrology, Department of Internal Medicine, University Medical Center Groningen, University of Groningen, Groningen, The Netherlands; 4 Department of Pharmacology and Therapy, Faculty of Medicine Universitas Airlangga, Surabaya, Indonesia; 5 Hospices Civils de Lyon, Hôpital Edouard Herriot, Service de Transplantation, Néphrologie et Immunologie Clinique, Lyon, France; 6 Department of Renal Medicine, Aarhus University Hospital, Aarhus, Denmark; 7 Department of Clinical Medicine, Aarhus University, Aarhus, Denmark; 8 Biomedical Sciences, Faculty of Health and Medical Science, University of Copenhagen, Copenhagen, Denmark; 9 Nordic Bioscience, Biomarkers and Research, Herlev, Denmark

**Keywords:** Nephrology, Kidney, Renal replacement therapy, Mechanisms of disease, Acute kidney injury, Chronic kidney disease, End-stage renal disease

## Abstract

Delayed graft function after kidney transplantation is common and increases morbidity and health care costs. There is evidence that endotrophin, a specific fragment of pro-collagen type VI, promotes the inflammatory response in kidney diseases. We tested the hypothesis that pretransplant endotrophin in kidney transplant recipients may be associated with the risk of delayed graft function. Pretransplant plasma endotrophin was assessed using an enzyme-linked immunosorbent assay in three independent cohorts with 806 kidney transplant recipients. The primary outcome was delayed graft function, i.e., the necessity of at least one dialysis session within one-week posttransplant. In the discovery cohort median pretransplant plasma endotrophin was higher in 32 recipients (12%) who showed delayed graft function when compared to 225 recipients without delayed graft function (58.4 ng/mL [IQR 33.4–69.0]; N = 32; vs. 39.5 ng/mL [IQR 30.6–54.5]; N = 225; P = 0.009). Multivariable logistic regression, fully adjusted for confounders showed, that pretransplant plasma endotrophin as a continuous variable was independently associated with delayed graft function in both validation cohorts, odds ratio 2.09 [95% CI 1.30–3.36] and 2.06 [95% CI 1.43–2.97]. Pretransplant plasma endotrophin, a potentially modifiable factor, was independently associated with increased risk of delayed graft function and may be a new avenue for therapeutic interventions.

## Introduction

Delayed graft function (DGF) after kidney transplantation describes the failure of the allograft to operate properly due to ischemia–reperfusion injury and a subsequent inflammatory response^1^. This complication can be observed in up to 40 percent of deceased donor kidney transplant recipients, prolongs patient hospitalization, increases morbidity and health care costs^2,3^. DGF is defined by the requirement for dialysis within the first week after transplantation^4^. A systematic review showed that risk prediction models for DGF commonly include recipient age and comorbidities^5^. Although several causes and mechanisms leading to DGF have been described in the last years, therapeutic or preventive options are still limited^6,7^.

Inflammatory mechanisms during ischemia and reperfusion together with immigration of cells, e.g., neutrophils, macrophages, and T lymphocytes, are hallmarks in the pathogenesis of DGF^7–9^. Factors linked to influx of cells into the graft after ischemia reperfusion injury could therefore be new targets for therapy and prevention. One factor that may promote this immigration of cells is endotrophin, which is a Pro-collagen type VI fragment^10^. Endotrophin is part of the inflammatory response. Endotrophin acts as a chemoattractant on macrophages, aggravates reperfusion injury, and increases inflammation^11–16^. Endotrophin levels were positively correlated with C-reactive protein in patients with chronic kidney disease^17^. Using a doxycycline-inducible mouse model, overexpression of endotrophin increased the inflammatory response in macrophages^13^. In vitro, endotrophin stimulated upregulation of proinflammatory genes including Toll-like receptor^13^.

Recent studies indicated that endotrophin was present in fibrotic kidneys but not in histologically normal kidneys^14^. A prospective study in patients with type 2 diabetes and microalbuminuria showed that doubling of plasma endotrophin levels significantly increased the risk for progression of kidney disease and deterioration of glomerular filtration rate^15^. Furthermore, studies in collagen type VI knockout mice showed limited ischemia-induced injury^16^. Since reperfusion injury is the main contributor to DGF, it is biologically plausible that pretransplant endotrophin may aggravate DGF. Endotrophin, as a potentially modifiable factor, may serve as a new avenue for therapeutic intervention and prevention of DGF.

We investigated whether pretransplant plasma endotrophin in incident kidney transplant recipients was associated with DGF after kidney transplantation.

## Materials and methods

### Study population and data source

Details about clinical cohorts, study design, and participants, detailed immunosuppression in discovery cohort and validation cohorts, sample collection and measurements of pretransplant plasma endotrophin as well as statistical analyses are given in Supplemental Methods.

In brief, this cohort study included incident kidney transplant recipients in three European transplant centers (Odense University Hospital, Odense, Denmark; University Medical Center Groningen, Groningen, The Netherlands; Aarhus University Hospital, Aarhus, Denmark). Written informed consent was obtained from all patients before entry into the studies. Exclusion criteria were age below 18 years or missing consent.

Details from the Molecular Monitoring after kidney transplantation (MoMoTx) study, the TransplantLines (TxL) Biobank and Cohort study, as well as “CONTEXT” study had been published previously^18–21^. The study protocols were in accordance with the ethical standards of the Declarations of Helsinki and Istanbul. Registration identifiers at ClinicalTrials.gov were NCT01515605, NCT03272841, and NCT01395719, respectively. The studies were approved by the local ethics committees (Den Videnskabsetiske Komite for Region Syddanmark, Projekt-ID: 20100098; METc 2014/077; and “CONTEXT” study^21^, respectively).

The primary outcome variable was DGF which was defined by United Network for Organ Sharing as dialysis within the first week after transplantation^22,23^. Need for dialysis was considered by the treating physicians according to local guidelines and best medical care after transplantation. Treating physicians were unaware of the pretransplant plasma endotrophin levels. Need for dialysis within the first week after transplantation was confirmed with chart review.

### Statistical analysis

Data were analyzed using non-parametric Mann–Whitney test, Fisher’s exact test or chi-square tests as appropriate. We performed receiver operating characteristic (ROC) analysis and determined a cutoff value using Youden index. We also used logistic regression to characterize the association between pretransplant plasma endotrophin, which was included as a continuous variable per increase in standard deviation and DGF. We adjusted for covariates using five models, with cumulative adjustment. All statistical tests were two-sided. Two-sided P-values less than 0.05 were considered to indicate statistical significance.

## Results

### Baseline characteristics of patients in the “MoMoTx” discovery cohort and determinants of DGF

In the “MoMoTx” discovery cohort we included 257 incident kidney transplant recipients before transplantation, 36 recipients (14%) had ABO-blood-type-incompatible living donors, 106 recipients (41%) had ABO-blood-type-compatible living donors, and 115 recipients (45%) had donation after brain death (DBD).

Table 1 summarizes the demographic and clinical characteristics of renal transplant recipients without and with DGF. In the “MoMoTx” discovery cohort DGF occurred in 32 out of 257 recipients (12%) for the entire group, 4 out of 36 (11%) for ABO-blood-type-incompatible living donor transplants, 11 out of 106 (10%) for ABO-blood-type-compatible living donor transplants, and 17 out of 115 (15%) for DBD. Recipient age, recipient gender, cause of kidney disease, as well as donor age, number of HLA mismatches, and pretransplant plasma creatinine were similar between the groups. Recipients with longer dialysis vintage (months) were more likely to show DGF (Table 1).

**Table 1:** Demographic and clinical characteristics of renal transplant recipients in the discovery cohort and in the validation cohorts who were stratified according to posttransplant delayed graft function.

“MoMoTx” discovery cohort	All patients (N = 257)	Delayed graft function (N = 32)	No delayed graft function (N = 225)	P-value
Age of recipient (years)	52 (41–62)	53 (44–60)	52 (41–62)	0.89
Recipient gender male, N (%)	171 (67%)	24 (75%)	147 (65%)	0.32
Body weight (kg)	83 (71–94)	89 (60–101)	81 (73–93)	0.015
Systolic blood pressure (mmHg)	147 (130–160)	132 (112–156)	148 (131–160)	0.007
Diastolic blood pressure (mmHg)	85 (75–94)	81 (65–94)	85 (76–94)	0.05
**Cause of kidney disease, N (%)**				0.63
Glomerulo-nephritis	91 (35%)	13 (41%)	78 (35%)	
Diabetes mellitus	41 (16%)	6 (19%)	35 (15%)	
Hypertension	30 (12%)	5 (16)	25 (11%)	
Interstitial nephritis	15 (6%)	2 (6%)	13 (6%)	
Polycystic kidney disease	37 (14%)	4 (12%	33 (15%)	
Other/unknown	43 (17%)	2 (6%)	41 (18%)	
Dialysis vintage (months)	12 (2–25)	21 (9–48)	11 (2–24)	0.006
Age of the donor (years)	53 (45–63)	54 (46–71)	53 (45–63)	0.45
Number of HLA mismatches (range)^a^	3 (2–5)	4 (3–5)	3 (2–5)	0.23
Cold ischemic time (hours) for deceased donors only	14 (10–17)	16 (10–20)	14 (10–17)	0.34
AB0 blood type incompatibility, N (%)	36 (14%)	4 (13%)	32 (14%)	1.00
Plasma creatinine pretransplant, (µmol/L)^b^	716 (538–925)	848 (620–1005)	701 (530–888)	0.07

“TxL” validation cohort	All patients (N = 341)	Delayed raft function (N = 30)	No delayed graft function (N = 311)	P-value
Age of recipient (years)	56 (45–65)	58 (44–67)	56 (45–65)	0.38
Recipient gender male, N (%)	216 (63%)	21 (70%)	195 (63%)	0.43
Body weight (kg)	80 (70–90)	82 (68–98)	80 (70–90)	0.66
Systolic blood pressure (mmHg)	139 (127–153)	134 (117–151)	140 (127–154)	0.12
Diastolic blood pressure (mmHg)	81 (73–90)	78 (72–87)	81 (73–90)	0.26
**Cause of kidney disease, N (%)**				0.33
Glomerulo-nephritis	31 (9%)	2 (7%)	29 (9%)	
Diabetes mellitus	27 (8%)	5 (17%)	22 (7%)	
Hypertension	56 (16%)	2 (7%)	54 (17%)	
Interstitial nephritis	16 (5%)	1 (3%)	15 (5%)	
Polycystic kidney disease	66 (19%)	5 (17%)	61 (20%)	
Other/unknown	145 (43%)	15 (50%)	130 (42%)	
Dialysis vintage (months)	0 (0–18)	26 (12–41)	0 (0–15)	0.001
Age of the donor (years)	56 (46–64)	57 (44–67)	56 (47–64)	0.92
Number of HLA mismatches (range)^a^	3 (2–5)	4 (3–5)	3 (2–5)	0.19
Cold ischemic time (hours) for all donors (N = 341)	3 (3–4)	10 (6–14)	3 (3–4)	0.001
Cold ischemic time (hours) for deceased donors only (donation after brain death and donation after circulatory death; N = 74)^c^	11 (9–14)	13 (10–15)	11 (9–14)	0.32
AB0 blood type incompatibility, N (%)	25 (7%)	1 (3%)	24 (8%)	0.71
Plasma creatinine pretransplant, (µmol/L)^b^	578 (425–759)	673 (455–835)	568 (422–751)	0.26

**“CONTEXT” validation cohort**	All patients (N = 208)	Delayed graft function (N = 63)	No delayed graft function (N = 145)	P-value
Age of recipient (years)	59 (50–66)	59 (44–66)	59 (51–66)	0.22
Recipient gender male, N (%)	126 (60.6%)	38 (60.3)	88 (60.7)	0.96
Body weight (kg) (N = 193)	74 (66–82)	75 (64–85)	74.0 (68–81)	0.58
**Cause of kidney disease, N (%)**				0.002
Glomerulo-nephritis	49 (24%)	17 (27%)	32 (22%)	
Diabetes mellitus	25 (11%)	11 (17%)	14 (10%)	
Hypertension/vascular	21 (9%)	7 (11%)	14 (10%)	
Interstitial nephritis/reflux/obstructive	6 (3%)	1 (2%)	5 (3%)	
Polycystic kidney disease	43 (19%)	3 (5%)	40 (28%)	
Other/unknown	64 (29%)	24 (38%)	40 (28%)	
Age of the donor (years)	58 (52–65)	56 (50–63)	59 (52–67)	0.09
Number of HLA mismatches (range)^a^	3 (3–4)	3 (2–4)	3 (3–4)	0.51
Cold ischemic time (hours) for deceased donors (donation after brain death and donation after circulatory death; N = 206)^d^	13 (11–16)	15 (11–17)	13 (10–15)	0.01
Plasma creatinine pretransplant (µmol/L)^b^ (N = 206)	629 (494–753)	660 (547–785)	611 (485–747)	0.17

Delayed graft function was defined by at least one dialysis within the first week after transplantation.

Continuous data are presented as median (interquartile range). Categorical data are presented as numbers (percent). Groups containing continuous data were compared using Mann–Whitney test, whereas groups containing categorical data were compared using Fisher’s exact test or Chi-square test, as appropriate.

^a^HLA denotes human leukocyte antigen.

^b^To convert the values for creatinine to milligram per deciliter, divide by 88.4.

^c^Numbers retrieved in “TxL” validation cohort, all other data are obtained from the entire cohort.

^d^Numbers retrieved in “CONTEXT” validation cohort, all other data are obtained from the entire cohort.

### Association of pretransplant plasma endotrophin levels and DGF in the “MoMoTx” discovery cohort

Pretransplant plasma endotrophin levels in kidney transplant recipients are provided in Table 2. The median [interquartile range, IQR] plasma endotrophin in 257 kidney transplant recipients from the “MoMoTx” discovery cohort before transplantation was 40.6 ng/mL [30.8–58.3]. Recipients showing DGF had higher median [IQR] pretransplant plasma endotrophin when compared to recipients without DGF (58.4 ng/mL [33.4–69.0]; N = 32; vs. 39.5 ng/mL [30.6–54.5]; N = 225; P = 0.009 by non-parametric Mann–Whitney test). As shown in Table 2, recipients with ABO-compatible living donor transplantation showing DGF had higher median pretransplant plasma endotrophin when compared to recipients without DGF (68.1 ng/mL [63.4–75.2]; N = 11; vs. 42.4 ng/mL [31.8–60.0]; N = 95; P = 0.0004 by non-parametric Mann–Whitney test).

**Table 2:** Pretransplant plasma endotrophin in kidney transplant recipients in the discovery cohort and in the validation cohorts who were stratified according to delayed graft function.

	Pretransplant endotrophin (ng/mL)			P-value
	All patients	Delayed graft function	No delayed graft function	
**“MoMoTx” discovery cohort**				
All kidney transplant recipients	40.6 [30.8–58.3] (N = 257)	58.4 [33.4–69.0] (N = 32)	39.5 [30.6–54.5] (N = 225)	0.009
ABO-blood-type-incompatible living donor transplants	30.5 [23.8–37.0] (N = 36)	36.1 [26.1–43.2] (N = 4)	29.8 [22.4–36.8] (N = 32)	0.366
ABO-blood-type-compatible living donor transplants	47.0 [32.2–63.4] (N = 106)	68.1[63.4–75.2] (N = 11)	42.4 [31.8–60.0] (N = 95)	0.0004
Donation after brain death	43.3 [32.2–58.3] (N = 115)	50.5 [27.9–66.5] (N = 17)	41.7 [32.7–55.7] (N = 98)	0.505
**“TxL” validation cohort**				
All kidney transplant recipients	41.3 [29.7–60.1] (N = 341)	60.0 [41.9–100.9] (N = 30)	40.0 [29.0–58.6] (N = 311)	0.001
ABO-blood-type-incompatible living donor transplants	35.2 [26.8–62.4] (N = 25)	50.5 [50.5–50.5] (N = 1)	35.1 [26.4–63.7] (N = 24)	0.579
ABO-blood-type-compatible living donor transplants	37.7 [27.2–56.9] (N = 242)	62.0[37.4–121.9] (N = 7)	36.8 [27.2–56.6] (N = 235)	0.066
All kidney transplant recipients with deceased donors	55.7 [41.5–69.1] (N = 74)	60.0 [44.0–100.9] (N = 22)	52.4 [39.0–65.7] (N = 52)	0.036
Donation after brain death	56.6 [45.3–80.2] (N = 26)	96.1 [81.3–123.6] (N = 4)	55.2 [37.8–64.0] (N = 22)	0.007
Donation after circulatory death	52.4 [40.9–68.3] (N = 48)	58.3 [41.9–77.2] (N = 18)	50.5 [39.7–68.2] (N = 30)	0.302
**“CONTEXT” validation cohort**				
All kidney transplant recipients with deceased donors	50.6 [36.0–65.5] (N = 208)	56.6 [48.4–79.2] (N = 63)	45.3 [32.2–60.9] (N = 145)	0.0001
Donation after brain death	49.5 [35.8–65.0] (N = 192)	56.1 [50.1–77.5] (N = 51)	45.3 [32.3–60.7] (N = 141)	0.0001
Donation after circulatory death	55.0 [37.8–74.3] (N = 16)	57.8 [44.3–75.5] (N = 12)	45.5 [35.1–58.9] (N = 4)	0.450

Delayed graft function was defined by at least one dialysis within the first week after transplantation.

Continuous data are presented as median (interquartile range). Groups containing continuous data were compared using Mann–Whitney test.

For the entire “MoMoTx” discovery cohort the Receiver operating characteristic (ROC) analysis indicated that pretransplant plasma endotrophin predict DGF (Area under curve, 0.64 [95% CI 0.53–0.76]; P = 0.010). The cut-off level determined using Youden index was 61.65 ng/mL. 16 out of 51 renal transplant recipients (31%) with endotrophin higher than 61.65 ng/mL had DGF whereas only 16 out of 206 (8%) recipients with endotrophin lower than 61.65 ng/mL had DGF, which yielded an unadjusted odds ratio of 5.43 [95% CI 2.49–11.86]. Sensitivity, specificity, odds ratio, positive predictive value, negative predictive value as well as likelihood ratio are summarized in Table 3. For the entire “MoMoTx” discovery cohort the pretransplant plasma endotrophin cutoff level of 61.65 ng/mL had a negative predictive value of 0.92 [95% CI 0.88–0.95].

**Table 3:** Performance of pretransplant plasma endotrophin for delayed graft function in the discovery cohort and in the validation cohorts.

	DGF	No DGF	Total	OR [95% CI]	Sens. [95% CI]	Spec. [95% CI]	PPV [95% CI]	NPV [95% CI]	LHR
**“MoMoTx” discovery cohort**									
**MoMoTx entire cohort**				5.43 [2.49–11.86]	0.50 [0.32–0.68]	0.84 [0.79–0.89]	0.31 [0.19–0.45]	0.92 [0.88–0.95]	3.21
Above cutoff	16	35	51						
Below cutoff	16	190	206						
**All living donors**				7.16 [2.31–22.18]	0.60 [0.32–0,84]	0.83 [0.75–0.89]	0.29 [0.14–0.48]	0.95 [0.89–0.98]	3.46
Above cutoff	9	22	31						
Below cutoff	6	105	111						
**AB0 incompatible living donors**				2.33 [0.08–66.53]	0.00 [0.00–0.60]	0.97 [0.84–1.00]	0.00 [0.00–0.98]	0.89 [0.73–0.97	0.00
Above cutoff	0	1	1						
Below cutoff	4	31	35						
**AB0 compatible living donors**				19.86 [3.18–79.12]	0.82 [0.48–0.98]	0.78 [0.68–0.86]	0.30 [0.15–0.49]	0.97 [0.91–1.00]	3.70
Above cutoff	9	21	30						
Below cutoff	2	74	76						
**All deceased donors**^**a**^				4.58 [1.48–14.15	0.41 [0.18–0.67	0.87 [0.78–0.93]	0.35 [0.15–0.59]	0.89 [0.81–0.95]	3.10
Above cutoff	7	13	20						
Below cutoff	10	85	95						
**“TxL” validation cohort**									
**TxL entire cohort**				3.25 [1.51–7.00]	0.47 [0.28–0.66]	0.79 [0.74–0.83]	0.18 [0.12–0.25]	0.94 [0.92–0.96]	2.20
Above cutoff	14	66	80						
Below cutoff	16	245	261						
**TxL all living donors**				4.08 [0.99–16.86]	0.50 [0.16–0.84]	0.80 [0.75–0.85]	0.07 [0.04–0.14]	0.98 [0.96–0.99]	2.54
Above cutoff	4	51	55						
Below cutoff	4	208	212						
**TxL AB0 incompatible living donors**				0.95 [0.03–26.34]	0.00 [0.00–0.98]	0.75 [0.53–0.90]	0.00 [0.00–0.46	0.95 [0.74–1.00]	0.00
Above cutoff	0	5	6						
Below cutoff	1	18	19						
**TxL AB0 compatible living donors**				5.63 [1.22–26.05]	0.57 [0.18–0.90]	0.81 [0.75–0.86]	0.08 [0.04–0.15]	0.98 [0.96–0.99]	2.98
Above cutoff	4	45	49						
Below cutoff	3	190	193						
**TxL all deceased donors**				2.06 [0.73–5.77]	0.45 [0.24–0.68]	0.71 [0.57–0.83]	0.40 [0.26–0.56]	0.76 [0.67–0.82]	1.58
Above cutoff	10	15	25						
Below cutoff	12	37	49						
**TxL DBD**				22.85 [1.07–487.37]	1.00 [0.40–1.00]	0.73 [0.50–0.89]	0.40 [0.12–0.74]	1.00 [0.79–1.00]	3.67
Above cutoff	4	6	10						
Below cutoff	0	16	16						
**TxL DCD**				1.17 [0.33–4.08	0.33 [0.13–0.59]	0.70 [0.51–0.85]	0.40 [0.22–0.61]	0.64 [0.54–0.72]	1.11
Above cutoff	6	9	15						
Below cutoff	12	21	33						
**“CONTEXT” validation cohort**									
**CONTEXT entire cohort all deceased donors**				2.29 [1.22–4.32]	0.41 [0.29–0.54]	0.77 [0.69–0.83]	0.43 [0.34–0.54]	0.75 [0.71–0.79]	1.76
Above cutoff	26	34	60						
Below cutoff	37	111	148						
**CONTEXT DBD**				2.29 [1.16–4.52]	0.41 [0.28–0.56]	0.77 [0.69–0.83]	0.39 [0.30–0.50]	0.78 [0.74–0.82]	1.76
Above cutoff	21	33	54						
Below cutoff	30	108	138						
**CONTEXT DCD**				2.14 [0.17–27.10]	0.42 [0.15–0.72]	0.75 [0.19–0.99]	0.83 [0.45–0.97]	0.30 [0.17–0.47]	1.67
Above cutoff	5	1	6						
Below cutoff	7	3	10						

*“MoMoTx”* Molecular Monitoring after kidney transplantation, *DGR* delayed graft function, *OR* odds ratio, *Sens* sensitivity, *Spec* specificity, *PPV* positive predictive values, *NPV* negative predictive value, *LHR* likelihood ratio.

The cut-off level determined using Youden index was 61.65 ng/mL.

^a^All deceased donors in the “MoMoTx” discovery cohort were donation after brain death (DBD).

Furthermore, we also used pretransplant plasma endotrophin as a continuous variable per increase in standard deviation for logistic regression analyses which yielded an unadjusted odds ratio for DGF of 1.56 [95% CI 1.13–2.17]). We determined the additive impact of pretransplant plasma endotrophin to a model comprising pretransplant age, sex, body-mass index, and dialysis vintage. For the entire group adding pretransplant plasma endotrophin to this clinical model significantly increased net reclassification improvement (0.46 [95% CI 0.10–0.82]; P = 0.01). Spearman correlation of pretransplant plasma endotrophin was -0.18, 0.39, 0.06, and 0.54 for age, dialysis vintage (months), blood pressure, and pretransplant plasma creatinine, respectively. The variance of inflation was 1.22, 1.11, 1.13, and 1.36 for recipient age, dialysis vintage (months), blood pressure, and pretransplant plasma creatinine, respectively, thus precluding multicollinearity.

### Baseline characteristics of patients in the “TxL” validation and determinants of DGF

In the “TxL” validation cohort we included 341 incident kidney transplant recipients before transplantation, 25 recipients (7%) had ABO-blood-type-incompatible living donors, 242 recipients (71%) had ABO-blood-type-compatible living donors, 26 recipients (8%) had DBD, and 48 recipients (14%) had donation after circulatory death (DCD).

Table 1 also summarizes the demographic and clinical characteristics of renal transplant recipients without and with DGF.

In the “TxL” validation cohort DGF occurred in 30 out of 341 recipients (9%) for the entire group, 1 out of 25 (4%) for ABO-blood-type-incompatible living donor transplants, 7 out of 242 (3%) for ABO-blood-type-compatible living donor transplants, 4 out of 26 (15%) for DBD, and 18 out of 48 (38%) for DCD. Recipient age, recipient gender, cause of kidney disease, as well as donor age, number of HLA mismatches, and pretransplant plasma creatinine were similar between the groups. Recipients with longer dialysis vintage (months) were more likely to show DGF (Table 1).

### Confirmation of findings of pretransplant plasma endotrophin levels and DGF in the “TxL” validation cohort

The median [IQR] plasma endotrophin in 341 kidney transplant recipients from the “TxL” validation cohort before transplantation was 41.3 ng/mL [29.7–60.1] (Table 2). Recipients showing DGF had higher median [IQR] pretransplant plasma endotrophin when compared to recipients without DGF (60.0 ng/mL [41.9–100.9]; N = 30; vs. 40.0 ng/mL [29.0–58.6]; N = 311; P = 0.001 by non-parametric Mann–Whitney test).

As shown in Table 2, recipients with DBD showing DGF had higher median pretransplant plasma endotrophin when compared to recipients without DGF (96.1 ng/mL [81.3–123.6]; N = 4; vs. 55.2 ng/mL [37.8–64.0]; N = 22; P = 0.007 by non-parametric Mann–Whitney test).

We observed a positive correlation between pretransplant plasma endotrophin and C reactive protein in kidney transplant recipients (r = 0.13, p = 0.02).

Now we tested the predictive performance of the cut-off level of 61.65 ng/mL in the “TxL” validation cohort. 14 out of 80 renal transplant recipients (18%) with endotrophin higher than 61.65 ng/mL had DGF whereas only 16 out of 261 (6%) recipients with endotrophin lower than 61.65 ng/mL had DGF, which yielded an unadjusted odds ratio of 3.25 [95% CI 1.51–7.00] (Table 3). For the entire “TxL” validation cohort the pretransplant plasma endotrophin cutoff level of 61.65 ng/mL had a negative predictive value of 0.94 [95% CI 0.92–0.96].

**Figure 1:**
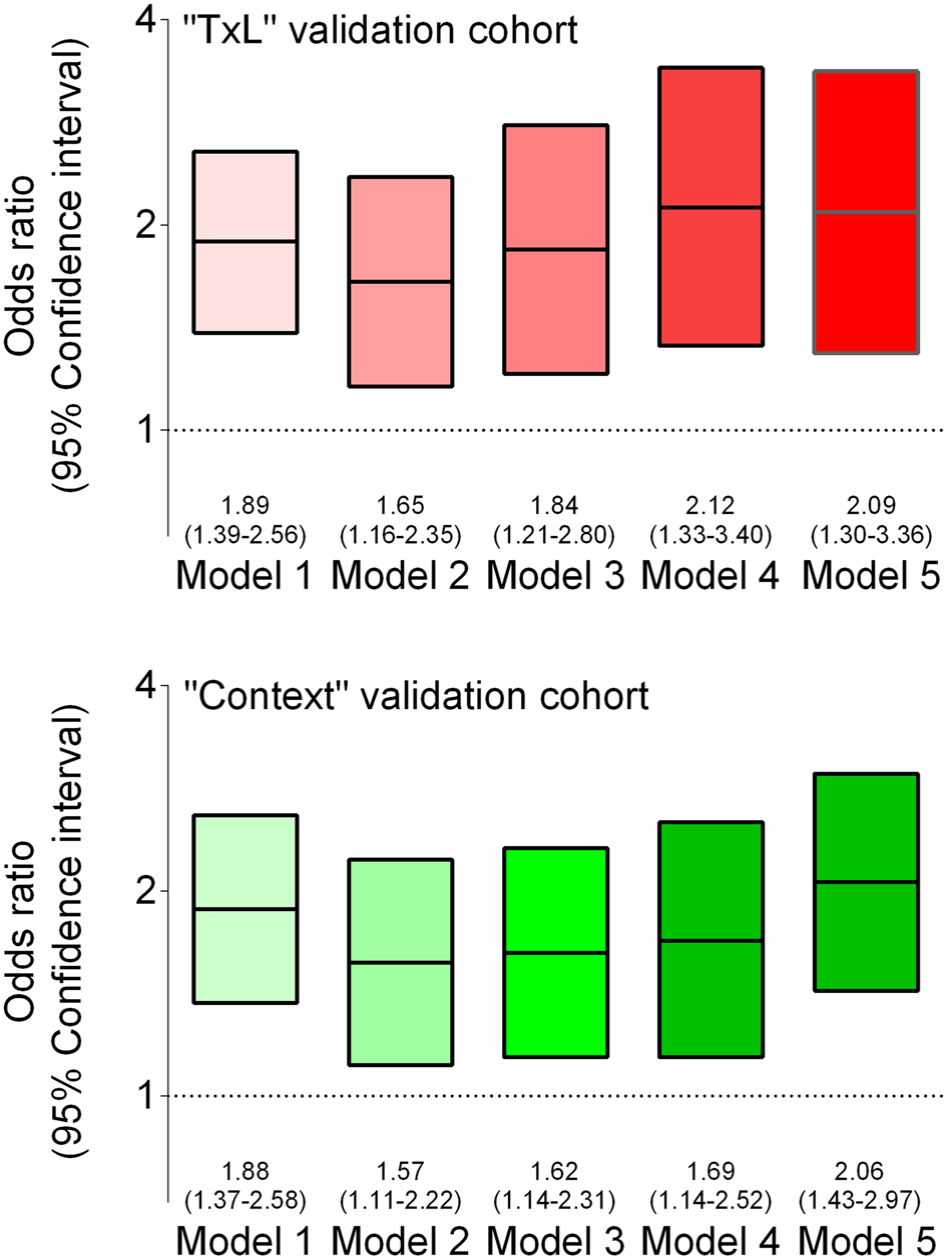
Risk of delayed graft function in the two independent validation cohorts, “TxL” and “CONTEXT”. The graphs show the odds ratios and 95% confidence intervals using logistic regression to characterize the association between continuous pretransplant plasma endotrophin per increase in standard deviation and delayed graft function. Model 1 was unadjusted. Model 2 was adjusted for age, sex, race, and dialysis vintage (months). Model 3 was further cumulative adjusted for blood pressure and transplant type. Model 4 was further cumulative adjusted for pretransplant plasma creatinine. Model 5 was further cumulative adjusted for cold ischemic time. Adjustment for “CONTEXT” validation cohort did not include race, dialysis vintage (months), and blood pressure.

Furthermore, we also used pretransplant plasma endotrophin levels as a continuous variable per increase in standard deviation for logistic regression analyses which yielded an unadjusted odds ratio for DGF of 1.89 [95% CI 1.39–2.56]). The association between pretransplant plasma endotrophin and DGF remained independent of adjustment for age, sex, race, and dialysis vintage (model 2; adjusted odds ratio 1.65; 95% CI 1.16–2.35), further cumulative adjustment for blood pressure and transplant type (model 3; adjusted odds ratio 1.84; 95% CI 1.21–2.80), further cumulative adjustment for pretransplant plasma creatinine (model 4; adjusted odds ratio 2.12; 95% CI 1.33–3.40), and further cumulative adjustment for cold ischemic time (model 5; adjusted odds ratio 2.09; 95% CI 1.30–3.36; Fig. 1). Further cumulative adjustment for diabetes mellitus showed (model 6) an adjusted odds ratio, 2.11; 95% CI 1.31–3.42. In the subgroup with recipients with ABO-compatible living donor transplantation pretransplant plasma endotrophin yielded an odds ratio for DGF of 1.77 [95% CI 1.06–2.96]. In the subgroup with recipients with deceased donors pretransplant plasma endotrophin yielded an odds ratio for DGF of 2.01 [95% CI 1.15–3.52].

### Baseline characteristics of patients in the “CONTEXT” validation cohort and determinants of DGF

In the “CONTEXT” validation cohort we included 208 incident kidney transplant recipients before transplantation, 192 recipients (92%) had DBD, and 16 recipients (8%) had DCD.

In the “CONTEXT” validation cohort DGF occurred in 63 out of 208 recipients (30%) for the entire group, 51 out of 192 (27%) for DBD, and 12 out of 16 (75%) for DCD.

Table 1 also summarizes the demographic and clinical characteristics of renal transplant recipients without and with DGF. Recipient age, recipient gender, as well as donor age, number of HLA mismatches, and pretransplant plasma creatinine were similar between the groups (Table 1).

### Confirmation of findings of pretransplant plasma endotrophin levels and DGF in the “CONTEXT” validation cohort

The median [IQR] plasma endotrophin in 208 kidney transplant recipients with deceased donors from the “CONTEXT” validation cohort before transplantation was 50.6 ng/mL [36.0–65.5] (Table 2). Recipients showing DGF had higher median [IQR] pretransplant plasma endotrophin when compared to recipients without DGF (56.6 ng/mL [48.4–79.2]; N = 63; vs. 45.3 ng/mL [32.2–60.9]; N = 145; P = 0.0001 by non-parametric Mann–Whitney test).

As shown in Table 2, recipients with DBD showing DGF had higher median pretransplant plasma endotrophin when compared to recipients without DGF (56.1 ng/mL [50.1–77.5]; N = 51; vs. 45.3 ng/mL [32.3–60.7]; N = 141; P = 0.0001 by non-parametric Mann–Whitney test).

Next, we tested the predictive performance of the cut-off level of 61.65 ng/mL in the “CONTEXT” validation cohort. 26 out of 60 renal transplant recipients (43%) with endotrophin higher than 61.65 ng/mL had DGF whereas only 37 out of 148 (25%) recipients with endotrophin lower than 61.65 ng/mL had DGF, which yielded an unadjusted odds ratio of 2.29 [95% CI 1.22–4.32] (Table 3). For the entire “CONTEXT” validation cohort the pretransplant plasma endotrophin cutoff level of 61.65 ng/mL had a negative predictive value of 0.75 [95% CI 0.71–0.79].

Furthermore, we also used pretransplant plasma endotrophin levels as a continuous variable per increase in standard deviation for logistic regression analyses which yielded an unadjusted odds ratio for DGF of 1.88 [95% CI 1.37–2.58]). The association between pretransplant plasma endotrophin and DGF remained independent of adjustment for age and sex (model 2; adjusted odds ratio 1.57; 95% CI 1.11–2.22), further cumulative adjustment for transplant type (model 3; adjusted odds ratio 1.62; 95% CI 1.14–2.31), further cumulative adjustment for pretransplant plasma creatinine (model 4; adjusted odds ratio 1.69; 95% CI 1.14–2.52), and further cumulative adjustment for cold ischemic time (model 5; adjusted odds ratio 2.06; 95% CI 1.43–2.97; Fig. 1).

The “CONTEXT” study indicated that the intervention, i.e., the repetitive inflation and deflation of a cuff around the thigh of the recipient, did not produce any effect on early kidney transplant functions^21^. In line with these results the association between pretransplant plasma endotrophin and DGF was observed in 107 recipients who had the sham procedure (1.97 [95% CI 1.22–3.17]) as well in 101 recipients who had the intervention (1.83 [95% CI 1.21–2.76]).

**Figure 2:**
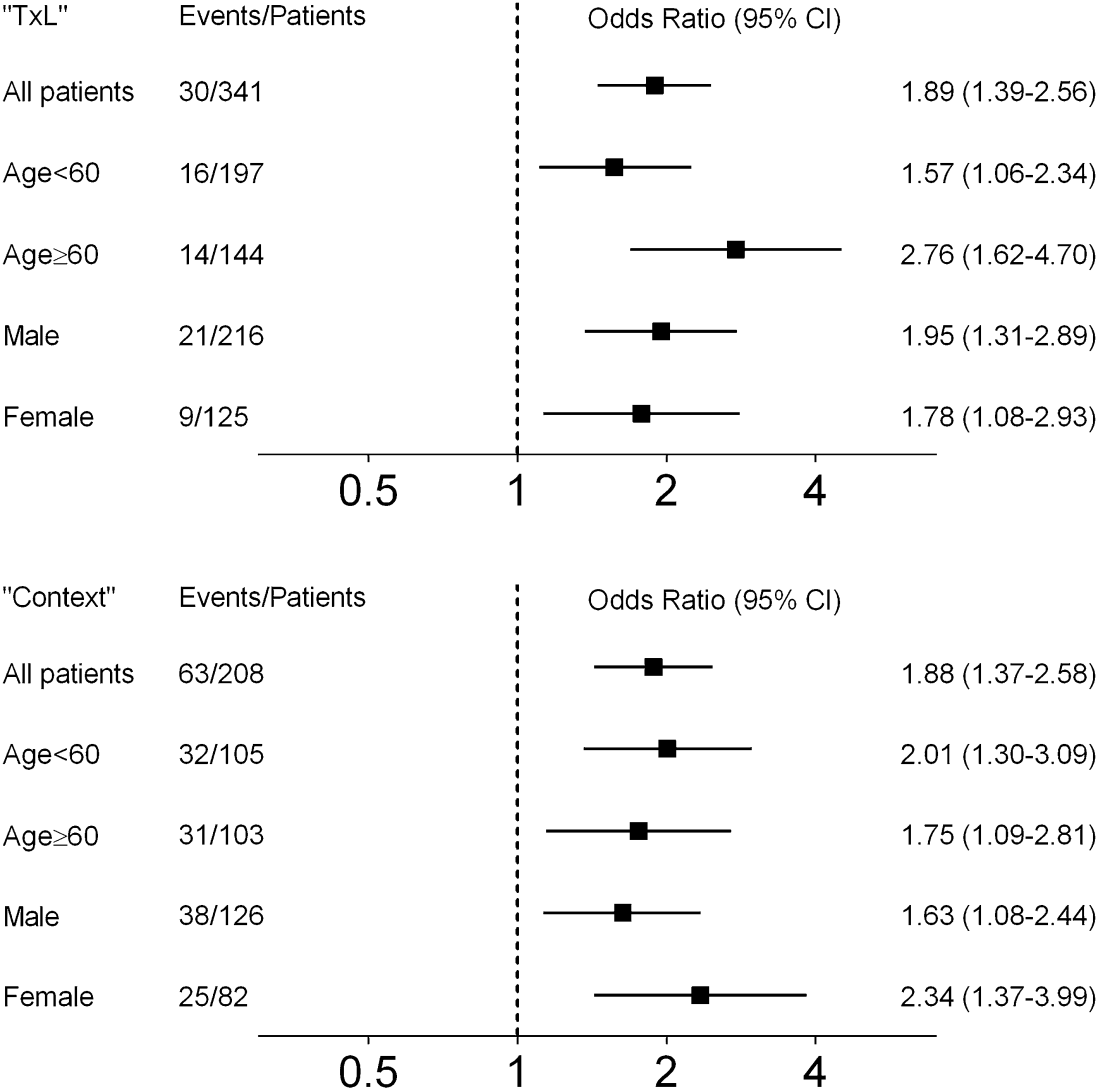
The graphs show the odds ratios and 95% confidence intervals (95% CI) for delayed raft function in the validation cohorts “TxL” and “CONTEXT”, using logistic regression to characterize the association between continuous pretransplant plasma endotrophin per increase in standard deviation and delayed graft function according to subgroup in the unadjusted analysis (Model 1). Subgroups included: Age < 60 years; Age ≥ 60 years; Male recipient; Female recipient.

Figure 2 shows odds ratios and 95% CI for DGF using logistic regression for several subgroups from the “TxL” and “CONTEXT” validation cohorts. These subgroup analyses showed that pretransplant plasma endotrophin was associated with DGF in male as well as female recipients, and in recipients younger as well as older than 60 years.

## Discussion

We investigated the association between pretransplant endotrophin and DGF after incident kidney transplantation with recipients who obtained living as well as deceased donors. We found that higher pretransplant plasma endotrophin was associated with subsequent DGF in three independent prospective cohorts. Determination of pretransplant plasma endotrophin in recipients may help to reduce delayed graft function after kidney transplant, since higher pretransplant plasma endotrophin levels may be attributed to worse outcome. There is an urgent need to reduce DGF because it is associated with longer hospitalizations and kidney rejection^23,24^. A systematic review and meta-analysis indicated that compared to patients without DGF, patients with DGF had a 41% increased risk of graft loss at 3.2 years of follow-up^24^. Tapiawala et al. reported that 23% out of 50,246 kidney transplant recipients showed DGF^24^. Compared with recipients without DGF, recipients with DGF were significantly more likely to die with a functioning graft^25^.

Some markers including urinary neutrophil gelatinase-associated lipocalin, kidney injury molecule-1, and calprotectin, have been investigated posttransplant to indicate DGF. They are early markers whose levels rise only after kidney injury has occurred^26–28^. Endotrophin can clearly be distinguished from these markers which allow early detection, but not pretransplant association with DGF. Notably, the association between pretransplant plasma endotrophin and DGF persisted independent of adjustment for clinically important covariates, including age, sex, dialysis vintage (months) pretransplant plasma creatinine, and cold ischemic time.

We observed the association of pretransplant plasma endotrophin with DGF in the discovery cohort. It should be noted that recipients’ factors including diabetes as well as donor age were similar in recipients who had DGF and no-DGF. We performed Receiver operator characteristics (ROC) analyses to define a cut-off in the discovery cohort and tested that cut-off value in the validation cohorts. We found consistent results in all cohorts with a broad spectrum of incident kidney transplant recipients. The size of the “TxL” validation cohort was larger, and the number of deceased donor transplants was higher in the “CONTEXT” validation cohort compared with the discovery cohort. Furthermore, using pretransplant plasma endotrophin levels as a continuous variable per increase in standard deviation for logistic regression analyses yielded to fully adjusted odds ratio for DGF of 2.06 and 2.09 in the validation cohorts, respectively. These findings indicate that pretransplant plasma endotrophin is associated with the development of DGF in real-world transplant settings, and that this finding is independently replicable. Notably, our fully adjusted models showed that pretransplant plasma endotrophin levels were associated with DGF, whereas characteristics including age, pretransplant plasma creatinine, and cold ischemic time were not associated with DGF. We observed the association of pretransplant plasma endotrophin with DGF when looking at a clinically relevant cut-off level as well as when using pretransplant plasma endotrophin as a continuous variable per increase in standard deviation.

The primary outcome, DGF, was defined according to well-established criteria of the United Network for Organ Sharing^22,23^. Notably, the incidence of DGF in the cohorts was between approximately 3% in living donor transplant recipients and approximately 30% in deceased donor transplant recipients, respectively, which is in line with previous reports^22^ indicating that our cohorts are representative for kidney transplant recipients in general.

In our validation cohorts we showed that higher pretransplant plasma endotrophin was associated with DGF in recipients with DBD grafts, but not with DCD grafts. These findings are in line with results from a recent meta-analysis confirming that DCD grafts per se are much more susceptible to DGF than DBD grafts which is mainly related to the donation process^29^. That implies that properties of the kidney transplant recipients have less impact in DCD grafts. Furthermore, our study gave strong evidence that properties of the kidney transplant recipients are of major importance with grafts of high quality and best donor processing, i.e., ABO-compatible living donor transplantation.

The present study showed that pretransplant plasma endotrophin was associated with acute allograft function. Furthermore, there is evidence that in patients with type 2 diabetes and microalbuminuria doubling of plasma endotrophin levels increased the risk for progression of kidney disease and deterioration of glomerular filtration rate^15^. Furthermore, plasma endotrophin was positively correlated with Banff interstitial fibrosis/tubular atrophy (IF/TA) scoring in transplant biopsies^30^. That may indicate that endotrophin may also be important for long term allograft outcome.

Standard induction therapy in our cohorts included different substances, e.g., basiliximab, anti-thymocyte globulin, and rituximab. Several previous studies indicated that different induction therapies showed similar rates of DGF^31,32^. Currently it is unknown whether plasma exchange or other therapies which are used for ABO-incompatible living donor transplantation may affect endotrophin levels. However, the present study had overall very small number of events in ABO-incompatible living donor transplantation, therefore future studies are necessary to evaluate further therapeutic opportunities.

Recent publications gave evidence that endotrophin is a potentially modifiable factor. Plasma endotrophin was determined in samples which had been obtained in a multicentre, open-label, randomised trial, which compared the 52-week treatment with the glucagon-like peptide-1 receptor agonist, dulaglutide, to insulin glargine in 329 patients with type 2 diabetes and moderate-to-severe chronic kidney disease^33^. Compared with insulin glargine, treatment with dulaglutide significantly attenuated the rise of plasma endotrophin^33^. In an animal model, the administration of the thiazolidinedione, rosiglitazone, to FP365PyMT mice reduced endotrophin transcripts as well as endotrophin proteins in tissue^34^.

## Supplementary Information

Supplementary Information.

## Data Availability

Access to data will be granted, on condition that researchers have appropriate ethical permission and sign the appropriate Material Transfer Agreement form.

## Abbreviations

DGF: Delayed graft function
DBD: Donation after brain death
DCD: Donation after circulatory death
Endotrophin: Pro-collagen type VI fragment
OR: Odds ratio
95% CI: 95 Percent confidence interval
