# Supplementary material for: Pretransplant endotrophin predicts delayed graft function after kidney transplantation
Source: Sci Rep. 2022 Mar 8;12:4079. doi: 10.1038/s41598-022-07645-y (PMC8904626; doi:10.1038/s41598-022-07645-y)
Supplement: Supplementary file 1 — Supplementary Information. [file 41598_2022_7645_MOESM1_ESM.docx]

# Pretransplant endotrophin predicts delayed graft function after kidney transplantation

**Martin Tepel^1^*, Firas F. Alkaff^2,3^*, Daan Kremer^2^, Stephan J.L. Bakker^2^, Olivier Thaunat^4^, Subagini Nagarajah^1^, Qais Saleh^1^, Stefan P. Berger^2^, Jacob van den Born^2^, Nicoline V. Krogstrup^5^, Marie B. Nielsen^5^, Rikke Nørregaard^6^, Bente Jespersen^5^, Nadja Sparding^7,8^, Federica Genovese^8^, Morten A. Karsdal^8^, and Daniel G.K. Rasmussen^8^**

**Supplemental Methods**

**Clinical cohorts, study design, and participants**

This cohort study included incident kidney transplant recipients in three European transplant centers (Odense University Hospital, Odense, Denmark; University Medical Center Groningen, Groningen, The Netherlands; Aarhus University Hospital, Aarhus, Denmark) after written informed consent was obtained before entry into the study.

In the “MoMoTx” discovery cohort samples were prospectively collected from Nov 6, 2013 until Jan 12, 2020 in the ongoing Molecular Monitoring after kidney transplantation (MoMoTx) study at Odense University Hospital, Denmark. Details from the “MoMoTx” study had been published previously^18,19^. The study protocol was in accordance with the ethical standards of the Declarations of Helsinki and Istanbul and its registration identifier at ClinicalTrials.gov is NCT01515605. The study was approved by the local ethics committee (Den Videnskabsetiske Komite for Region Syddanmark, Projekt-ID: 20100098). The “MoMoTx” discovery cohort comprised recipients from ABO-blood-type-incompatible living donor transplants, ABO-blood-type-compatible living donor transplants, and from deceased donors, i.e., donation after brain death (DBD). Written informed consent was obtained from all patients before entry into the study. Exclusion criteria were age below 18 years or missing consent.

In the “TxL” validation cohort samples were prospectively collected from Jan 1, 2015 until 2020 in the ongoing TransplantLines (TxL) Biobank and Cohort study at the University Medical Centre Groningen, The Netherlands. Details from the “TxL” study have been published previously^20^. The study protocol was in accordance with the ethical standards of the Declarations of Helsinki and Istanbul and its registration identifier at ClinicalTrials.gov is NCT03272841. The study was approved by the institutional ethical review board (METc 2014/077). The “TxL” validation cohort comprised recipients from ABO-blood-type-incompatible living donor transplants, ABO-blood-type-compatible living donor transplants, and from deceased donors, i.e., DBD as well as donation after circulatory death (DCD). Written informed consent was obtained from all patients before entry into the study. Exclusion criteria were age below 18 years or missing consent.

In the “CONTEXT” validation cohort samples were prospectively collected from a multicenter, randomized, controlled clinical trial in transplantation centers in Aarhus, Denmark; Gothenburg, Sweden; Groningen, The Netherlands; and Rotterdam, The Netherlands, from June 12, 2011 until Dec 28, 2014, hence, there was no overlap with the “TxL” cohort with recipients from The Netherlands. Results from the “CONTEXT” study had been reported previously, indicating that the intervention, i.e., the repetitive inflation and deflation of a cuff around the thigh of the recipient, did not produce any effect on early kidney transplant functions^21^. In the CONTEXT validation cohort 225 patient were initially included in the study. Three patients were then excluded^21^ and further 14 patients were excluded from the present study due to missing plasma samples in 12 patients and graftectomy first week in two patients, leaving 208 incident kidney transplant recipients eligible for the present study. The study protocol was in accordance with the ethical standards of the Declarations of Helsinki and Istanbul and its registration identifier at ClinicalTrials.gov is NCT01395719. The study was approved by the local ethical committees and the data protection agencies in each country. The “CONTEXT” validation cohort comprised recipients from deceased donors, i.e., DBD and, in centers from The Netherlands, in addition DCD. Written informed consent was obtained from all patients before entry into the study. Exclusion criteria were age below 18 years or missing consent.

The baseline characteristics of donors and recipients and information on organ procurement were obtained from electronic medical records. Data were pseudo anonymized and collected for the respective cohorts in accordance with local regulations. The authors had full access to data and take responsibility for the completeness and accuracy of the data and for the integrity of the analysis.

All transplantations were performed with negative complement-dependent cytotoxicity crossmatches. Immunosuppressive therapies were administered according to local therapeutic guidelines. Immunosuppression consisted of various combinations of basiliximab, rituximab, prednisolone, anti-thymocyte globulin, calcineurin inhibitors, and antiproliferative mycophenolate according to recipients’ immunological risks and clinical transplant protocols.

**Detailed immunosuppression in discovery cohort and validation cohorts**

In the “MoMoTx” discovery cohort all patients were treated with the calcineurin inhibitor, tacrolimus (0.25 milligram per kilogram body weight daily). All patients were treated with anti-proliferative mycophenolate (i.e., mycophenolate mofetil, 1.000 milligram twice daily, or mycophenolate sodium, 720milligram twice daily). Induction therapies included anti-interleukin-2 receptor antibody (basiliximab, 20 milligram pretransplant and 3 days post-transplant), anti-B cell cluster of differentiation CD20 antibody (rituximab, 375 milligram per square meter, 4 weeks pretransplant), prednisolone (25 milligram daily), and T cell–depleting anti-thymocyte globulin (thymoglobuline, 1.5 milligram per kilogram body weight pretransplant. ABO-incompatible living donor transplant recipients additionally received rituximab and immunopheresis. All recipients received prophylaxis with either acyclovir at 200 milligram daily or valganciclovir at 450 milligram daily, respectively.

In the “TxL” validation cohort, standard immunosuppressive treatment at transplantation consisted of calcineurin inhibitor tacrolimus (0.15 milligram per kilogram bodyweight per day), the proliferation inhibitor, mycophenolate mofetil (2000 milligram daily), and prednisolone (20 milligram pretransplant and 3 days post-transplant). In case of intolerance, either the calcineurin inhibitor cyclosporine A (4 milligram per kilogram bodyweight daily) or the proliferation inhibitor azathioprine (2 milligram per kilogram bodyweight daily) were usually considered as alternatives. Standard induction therapy included combinations of anti-interleukin-2 receptor antibody basiliximab (20 milligram pretransplant and 3 days post-transplant). T cell–depleting anti-thymocyte globulin (thymoglobuline, 1.5 milligram per kilogram body weight pretransplant), or alemtuzumab (30 milligram) were used as alternatives in case of highly immunized recipients. ABO-incompatible living donor transplant recipients additionally received rituximab and immunopheresis. All recipients that tested positive for cytomegalovirus antibodies or received a kidney from a cytomegalovirus positive donor received prophylaxis with valganciclovir at 450 milligram daily.

In the “CONTEXT” validation cohort the immunosuppressive regimen was based on intravenous basiliximab (20 milligram pretransplant and 3 days post-transplant) and oral maintenance therapy based on calcineurin inhibitors (e.g., tacrolimus, 0.15 milligram per kilogram body weight daily), mycophenolate mofetil (either 1000 or 750 milligram twice daily) and prednisolone according to local guidelines.

**Determination of DGF after transplantation**

The primary outcome variable was DGF which was defined by United Network for Organ Sharing as dialysis within the first week after transplantation^22,23^. Need for dialysis was considered by the treating physicians according to local guidelines and best medical care after transplantation. Treating physicians were unaware of the pretransplant plasma endotrophin levels. Need for dialysis within the first week after transplantation was confirmed with chart review.

**Sample collection and measurements of pretransplant plasma endotrophin**

Blood samples were collected pretransplant in tubes with heparin or ethylenediaminetetraaceticacid. Within 2 hours after obtaining blood samples, plasma was prepared by centrifugation, aliquoted in 1000 µL vials and stored at -70°C until determination.

The enzyme-linked immunosorbent assay (ELISA) used to measure endotrophin in plasma was developed at Nordic Bioscience (Herlev, Denmark). The ELISA detects an active fragment of collagen type VI, which is released upon deposition in the extracellular matrix. The epitope recognized by the monoclonal antibody, KPGVISVMGT, is found at amino acid position 3168-3177. The detection range of the assay was 0.6-97.2 ng/ml, and the intra- and inter-assay variations were 4.0 and 12.7 percent, respectively. There is no cross-reactivity with other proteins or substances. Experienced technicians who were unaware of the clinical data measured plasma endotrophin using a standardized ELISA protocol.

**Statistical analysis**

Continuous data are presented as median and interquartile range (IQR). Frequency counts were calculated for categorical data. Contingency tables were analyzed using Fisher’s exact test or chi-square tests. To compare kidney transplant recipients between groups, we used the non-parametric Mann-Whitney test for continuous variables and Fisher’s exact test or chi-square tests for categorical variables.

We performed receiver operating characteristic (ROC) analysis to detect the accuracy of endotrophin to predict delayed allograft function. The cut-off level was determined using Youden index.

We used logistic regression to characterize the association between pretransplant plasma endotrophin, which was included as a continuous variable per increase in standard deviation and DGF as primary outcome. We adjusted for covariates using six models, with cumulative adjustment. **Model 1** was unadjusted. **Model 2** was adjusted for age, sex, race, and dialysis vintage (months). **Model 3** was additionally adjusted for blood pressure and transplant type. **Model 4** was additionally adjusted for pretransplant plasma creatinine. **Model 5** was additionally adjusted for cold ischemic time. **Model 6** was additionally adjusted for diabetes mellitus. The performance of the model was evaluated using a Hosmer-Lemeshow goodness-of-fit test and a P>0.05.

Adjustment in the “CONTEXT” validation cohort did not include race, dialysis vintage (months), and blood pressure because these covariates were not available. To evaluate whether the association between pretransplant plasma endotrphin and DGF was modulated by the sham procedure or intervention used in “CONTEXT”, i.e., the repetitive inflation and deflation of a cuff around the thigh of the recipient, we used logistic regression models including pretransplant plasma endotrophin and the intervention.

We assessed the impact of pretransplant plasma endotrophin in addition to a model comprising pretransplant clinical parameters by determining net reclassification improvement. Exact confidence intervals for net reclassification improvement were provided.

We determined Spearman correlation for pretransplant plasma endotrophin and recipients’ clinical and laboratory characteristics. We explored multicollinearity of pretransplant plasma endotrophin and recipients’ characteristics in a multiple regression model by determination of the variance inflation factor. A variance inflation factor less than 5 was considered to preclude multicollinearity.

Data were analyzed using GraphPad prism software (version 6.0, GraphPad Software, La Jolla, CA, USA), R with the package PredictABEL (version 1.2-2), and IBM SPSS Statistics (version 25; [www.ibm.com/products/spss-statistics](http://www.ibm.com/products/spss-statistics)). All statistical tests were two-sided. Two-sided P-values less than 0.05 were considered to indicate statistical significance.
